# Comparative Study of Metabolomic Profile and Antioxidant Content of Adult and In Vitro Leaves of *Aristotelia chilensis*

**DOI:** 10.3390/plants11010037

**Published:** 2021-12-23

**Authors:** Karina Andrea Crisóstomo-Ayala, Ana Belén Sabater-Jara, Claudia Pérez Manriquez, Federico Ferreres, Ángel Gil-Izquierdo, Maria Ángeles Pedreño, Martha Hernández de la Torre, Manuel Sanchez-Olate, Darcy Graciela Ríos Leal

**Affiliations:** 1 Centro de Biotecnología, Facultad de Ciencias Forestales, Universidad de Concepción, Victoria 631, Barrio Universitario, Casilla 160-C-Correo 3, Concepcion 4030000, Chile; marhernandez@udec.cl (M.H.d.l.T.); msanche@udec.cl (M.S.-O.); drios@udec.cl (D.G.R.L.); 2 Department of Plant Biology, Faculty of Biology, University of Murcia, 30100 Murcia, Spain; anabelen.sabater@um.es (A.B.S.-J.); mpedreno@um.es (M.Á.P.); 3 Departamento de Botánica, Facultad de Ciencias Naturales y Oceanográficas, Universidad de Concepción, Barrio Universitario, Casilla 160-C, Concepcion 4030000, Chile; claudiaperez@udec.cl; 4 Department of Food Technology and Nutrition, Molecular Recognition and Encapsulation (REM) Group, Campus Los Jerónimos, Universidad Católica de Murcia, UCAM, s/n, 30107 Murcia, Spain; fferreres@ucam.edu; 5 Research Group on Quality, Safety and Bioactivity of Plant Foods, Department of Food Science and Technology, CEBAS (CSIC), P.O. Box 164, 30100 Murcia, Spain; angelgil@cebas.csic.es

**Keywords:** *A. chilensis*, phenol, HPLC, adult leaves, in vitro leaves

## Abstract

This work aimed to identify the bioactive compounds present in adult maqui (*Aristotelia chilensis*) leaves from different stages of development and seasons of the year and compare them with leaves obtained from maqui plants grown in vitro. The qualitative and quantitative analysis of maqui leaf extracts by HPLC-DAD-ESI-MS^n^ showed the presence of different polyphenolic compounds classified into galloyl and caffeoyl quinic acids, ellagitannins and ellagic acid- and flavonoid-derivatives. In general, the total phenolic content of the in vitro samples was higher than that of ex vitro samples, whereas the total flavonoid content was higher in winter basal leaves. Additionally, the analysis by HPLC-MS showed that the extract from spring basal leaves was enriched in quercetin, catechin, kaempferol and 3-caffeoyl quinic acids, while in the in vitro leaves extract, quercetin was not present. As regards lipophilic compounds identified by GC/MS, the samples of in vitro leaves showed a high presence of α-tocopherol and β-sitosterol. In contrast, the samples of adult leaves presented a hight level of linolenic and linoleic acids. These results suggest that maqui leaves could be an excellent source of antioxidants and lipophilic compounds for many industries, such as the nutraceutical and pharmaceutical industries.

## 1. Introduction

*Aristotelia chilensis* is a perennial non-wood forest species belonging to the Elaeocarpaceae family. It is an endemic species of the sub-Antarctic forests of both Argentina and Chile. In a previous study, its cultivation extends from the region of Coquimbo to the Aysén region, including the Juan Fernández Islands [1]. *A. chilensis*, known as maqui, has been recognized for its beneficial effects on human health due to its antioxidant [2], anti-tumoral [3,4], cardioprotective [5], anti-inflammatory [6,7], anti-hemolytic [8] and anti-diabetics properties [9], as well as for its antiplatelet effect [10]. These biological activities are mainly attributed to its small fruits, containing high levels of polyphenols, particularly a wide variety of phenolic compounds such as phenolic acids, anthocyanins, pro-anthocyanidins and alkaloids [11].

For this reason, most of the studies carried out on maqui have been focused on using fruit extracts because they exhibit pharmacological activities of high relevance, mainly associated with the content of anthocyanins [11,12]. The anthocyanin profile in maqui fruit, which is abundant in delphinidin derivatives, has been characterized by different researchers [10,13]. These compounds are relevant because they are absorbed and metabolized in humans, circulating as sulphated and glucuronidated forms in the blood, which are accumulated in target tissues and excreted in the urine, and can be transported across the blood–brain barrier [14,15].

It is important to highlight that the content of flavonoids and other phenolic compounds depends on the intrinsic factors that affect the plant, such as the expression of specific genes, as well as the different geographical areas where maqui plants are cultivated [16]. Many environmental factors such as light, temperature, altitude, soil type, water, nutritional status, microbial interactions, pathogenesis, wounds, defoliation, growth regulators and seasonality, as well as the different agricultural techniques used, can influence the biosynthesis of these compounds in maqui plants [16].

On the other hand, given the beneficial health properties of these compounds, there is a growing interest in the knowledge of the metabolic profile of extracts of maqui fruits and leaves, and the high demand for these bioactive compounds has promoted the search for new alternative sources for the production of these compounds. Turchetti and Paz [17] described the presence of polyphenols and indole alkaloids such as aristoteline, aristotelinine, aristotelone, aristone, aristotelinone, aristoquinoline, makonine and hobartine, and their derivatives, amongst others, in maqui leaf extracts. Muñoz et al. [18] carried out a phytochemical characterization of leaf extracts, and detected their anti-inflammatory, analgesic and antioxidant activities. In addition, Céspedes et al. [19] carried out the first studies on the use of maqui leaf extracts in the treatment of Alzheimer’s disease. Nevertheless, few studies have considered the effects of seasonality and the state of development of maqui leaves in order to understand the season in which they have high levels of these specific bioactive phenolic compounds.

Furthermore, the growing market demand for these bioactive compounds can lead to over-harvesting of the different organs (mainly fruits and leaves) from endemic forest species, as is the case of maqui plants, which would make it an endangered species. In this way, when a scarce distribution in nature limits the supply of a bioactive compound, plant biotechnology can provide an alternative and sustainable system for its production. Phytochemical-producing green factories based on the use of the plant in vitro cultures have several advantages over plants grown in the field: a higher production of bioactive compounds with a lower use of natural sources and the obtention of uncontaminated plant material because in vitro cultures are maintained in an aseptic environment. Moreover, agricultural systems are not required, freeing up land for food production and drastically reducing the use of water [20].

Nevertheless, the main drawbacks of using in vitro culture techniques focus on the technological and economic viability of the process because it is necessary to find plant species or varieties from which in vitro plant cultures can be easily obtained and, once obtained, the main setback arises in the disinfection of the plant material that is to be introduced in vitro because contaminations by fungi, bacteria or viruses make it impossible to exploit this procedure [21]. Another possible drawback is the appearance of mutations or epigenetic variations due to stress situations or other factors, which can cause a decrease in yield in the production of plant biomass in vitro, the loss of productivity, slow growth or the problems of scaling. However, these variations can be minimized by adjusting the culture conditions in ranges that do not cause cellular stress [22].

Despite these reasons, in vitro tissue cultures can be considered a suitable and cutting-edge system for the sustainable production of phytochemicals that preserve their natural sources.

In this work, we investigate the influence of different stage of development and seasons on the *A. chilensis* leaf metabolite profile by analysing spectral data deduced by HPLC-DAD-ESI (Ion Trap)-MSn, HPLC-MS and GC-MS methods.

## 2. Results and Discussion

### 2.1. Qualitative Analysis of the Maqui Leaves

**Figure 1:**
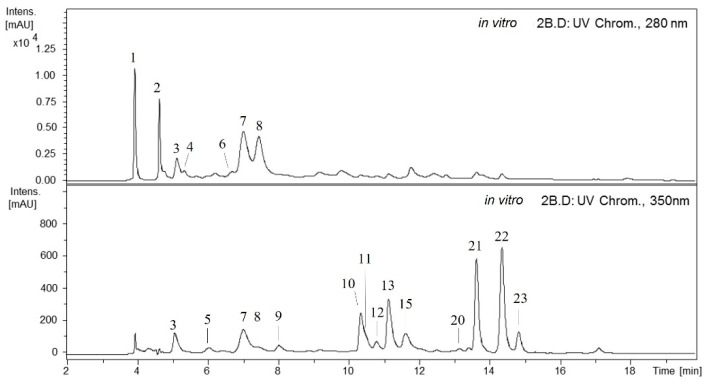
HPLC-DAD-ESI (Ion Trap)-MS^n^ (280, 350 nm) phenolic profile of methanolic extract from in vitro leaves of *A. chilensis*. Identity of compounds is shown in Table 1 and Table 2.

**Figure 2:**
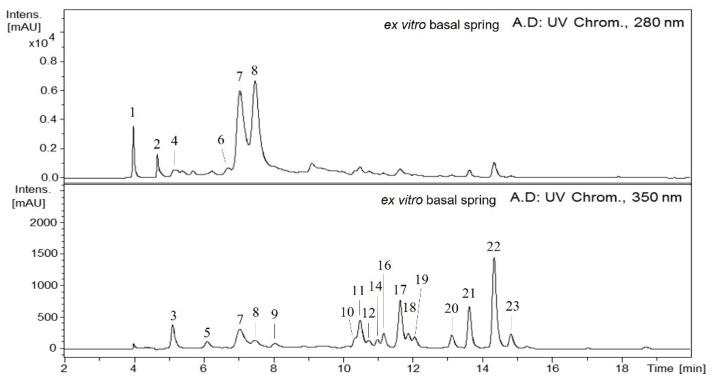
HPLC-DAD-ESI (Ion Trap)-MS^n^ (280, 350 nm) phenolic profile of methanolic extract from ex vitro basal spring leaves of *A. chilensis*. Identity of compounds is shown in Table 1 and Table 2.

Many phenolic compounds were detected in the methanolic extracts of *A. chilensis* leaves by HPLC-DAD-ESI (Ion Trap)-MS^n^ (Figure 1 and Figure 2). The compounds identified were classified into five main groups: galloyl acid derivatives, caffeoyl quinic acids, ellagitannins, ellagic acid derivatives and flavonoid derivatives.

#### 2.1.1. Galloyl Acid Derivatives

**Figure 3:**
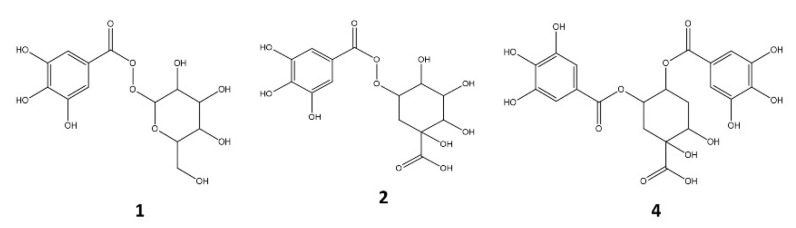
Structure of the main galloyl acid derivatives identified in *A. chilensis* leaf extracts.

Compounds **1**, **2** and **4** with Rt at 3.9, 4.6 and 5.3 min, respectively showed identical UV spectra (276 nm), and deprotonated molecular ions were at *m*/*z* 331, 343 and 495, respectively. In the MS fragmentation of **1,** the loss of a fragment at 162 amu (hexosyl radical) was observed, leading to an ion at *m*/*z* 169 (deprotonated molecular ion of gallic acid). Therefore, this compound was identified as galloyl-hexoside (**1**) [23]. In the MS fragmentations of **2,** a loss of 152 amu (radical galloyl) was detected to produce an ion at *m*/*z* 191 (deprotonated molecular ion of quinic acid), and thus, this compound was identified as galloyl quinic acid (**2**) [24]. The MS^2^ of **4** provided a deprotonated molecular ion higher than 2 (152 amu or increase with regards to 2) and showed an additional loss of 152 to obtain 343 (deprotonated molecular ion of 2). This compound provided a similar fragmentation pattern (MS^3^[495–343]^−^) than **2**, therefore, it was identified as di-galloyl quinic acid (**4**) (Figure 3) [24,25,26].

#### 2.1.2. Caffeoyl Quinic Acids

**Figure 4:**
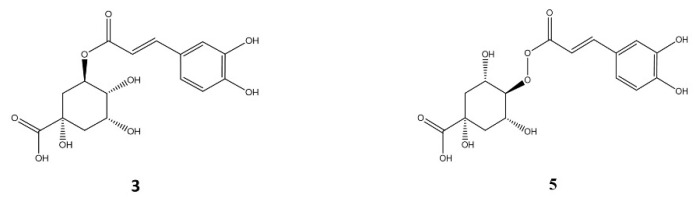
Structure of the main caffeoyl quinic acids identified in *A. chilensis* leaves extracts.

Compounds **3** and **5** (Rt 5.1 and 6.1 min, respectively) showed the same deprotonated molecular ions at *m*/*z* 353 and UV spectra (299sh, 324 nm) typical of cinnamoyl quinic acid structures. According to their MS fragmentations and their relative abundances (MS^2^(**3**): 191 (100%), 179 (50%); MS^2^(**5**): 179 (65%), 173 (100%)), and in accordance with Clifford et al. [27], it can be concluded that these compounds were 3-caffeoyl quinic acid (**3**) and 4-caffeoyl quinic acid (**5**) (Figure 4) [28,29].

#### 2.1.3. Ellagitannins

**Figure 5:**
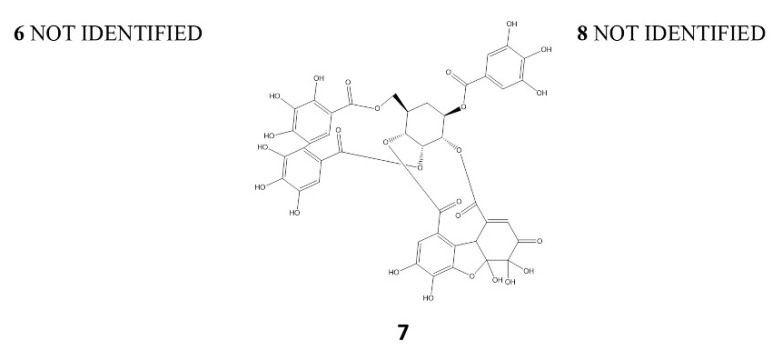
Structure of the main ellagitannins identified in *A. chilensis* leaf extracts. The structure 6 and 8 was not identified.

Compounds **6**, **7** and **8**, with Rt at 6.7, 7.0 and 7.4 min, respectively, presented the same UV spectra (278 nm) with an MS fragmentation of the deprotonated ion corresponding to ellagic acid (301 amu) [30,31]. Therefore, they were unknown polymeric structures composed of galloyl and hexahydroxydiphenoyl (HHDP) esterified with glucose. Peak **7** with [M-H]^−^ at *m*/*z* 951 and MS^2^: 933, 463, and 301 could be coincident with granatin B (galloyl-HHDP-DHHDP-hexoside) [31]; Peaks **6** and **8** showed the same mass and fragmentation pattern ([M-H]^−^ at *m*/*z*: 1109, MS^2^: 1049, 973, 935, 301), and their structures have not been identified (Figure 5).

#### 2.1.4. Ellagic Acid Derivatives

Compounds **9**, **10**, **12**, **13** and **15** showed UV spectra typical of ellagic acid derivatives (UV: 252, 305sh, 345sh, 364 nm) and their MS fragmentations; after the losses of 162 amu (**9**), 132 amu (**10** and **12**) and 146 amu (**13** and **15**), a base peak of ellagic acid was detected (301 amu) (Table 1) [32]. Therefore, these compounds were identified as ellagic acid-hexoside (**9**), ellagic acid-pentoside isomers (**10** and **12**) and ellagic acid-rhamnoside isomers (**13** and **15**) [32,33].

#### 2.1.5. Flavonoid Derivatives

Peaks **11**, **14** and **16–23** showed UV spectra characteristics of flavonoids (Table 2). Particularly, compounds **14**, **16**, **17**, **18** and **20** had UV spectra of quercetin substituted at the 3-*O* position (Table 2).

After the MS fragmentation, a base peak at *m*/*z* 301 (deprotonated quercetin) was observed. The deprotonated molecular ion of **14** and **16** at *m*/*z* 609 indicated that they were rhamno-hexosyl quercetin isomers, and the absence of other ions at the MS fragmentation spectra linked to the interglycosyl linkage suggests a bond 1–6 [34]. Therefore, both compounds should be quercetin-3-*O*-(6-rhamnosyl) hexosides isomers, labelled by elution order by reverse phase interaction as quercetin-3-*O*-(6-rhamnosyl) galactoside (**14**) and quercetin-3-*O*-(6-rhamnosyl) glucoside (**16**) [35]. Compounds **17**, **18** and **20** showed a deprotonated molecular ion of monoglycosides. Peaks **17** and **18** should be quercetin-3-*O*-hexosides isomers, which would agree with quercetin-3-*O*-galactoside (**17**) and quercetin-3-*O*-glucoside (**18**) [36], and quercetin-3-*O*-pentoside (**20**) (Table 2). Peak **11** presented a deprotonated molecular ion at *m*/*z* 615 with a loss of 152 amu after the MS fragmentation event (galloyl radical), and other ions at *m*/*z* 301 (deprotonated quercetin), which indicated that this compound was quercetin-3-*O*-(galloyl) hexoside. Its UV spectrum provided a maximum at 268 nm due to the overlaying of quercetin and gallic acid UV spectra [23]. Compound **19** had a monoglycoside mass and a UV spectrum of luteolin derivative. Its fragmentation showed a base peak at *m*/*z* 285 (deprotonated luteolin), confirming luteolin-7-*O*-hexoside [37,38]. In the MS fragmentation of **21** and **22**, an ion at *m*/*z* 285 as the base peak from their deprotonated aglycones was observed (tetrahydroxyflavone). Their UV spectra (Table 2) did not correspond neither with that of luteolin (5,7,3′,4′-tetrahydroxyflavone) nor kaempferol (3,5,7,4′-tetrahydroxyflavone). In fact, these UV spectra (maximum absorption peaks at 268 and 336 nm) were more similar to that of apigenin (5,7,4′-trihydroxyflavone), and the additional hydroxyl was not bound to ring A because the maximal absorption would be between 270 and 275 nm instead of 268; it could then be placed at the ring B 2′-position (5,7,2′,4′-tetrahydroxyflavone). Therefore, considering the comments above with regards to the glycosylated fraction of compounds **14/16**, **17/18**, **21** and **22**, they could be labelled as 5,7,2′,4′-tetrahydroxyflavone-*O*-(6-rhamnosyl) hexoside and 5,7,2′,4′-tetrahydroxyflavone-*O*-hexoside, respectively. Peak **23** showed a UV spectrum similar to that of luteolin, and its deprotonated molecular ion at *m*/*z* 659 with MS fragment ion at *m*/*z* 329 as the base peak (deprotonated trihydroxy-dimethoxyflavone) has not been properly identified.

### 2.2. Qualitative Differences of Phenolics from In Vitro and Ex Vitro Samples

The screening of phenolic compounds from methanolic extracts obtained from in vitro (samples: 1 and 2) and ex vitro (samples: spring apical, winter apical, spring basal and winter basal) by HPLC–DAD–ESI/MS^n^ showed a HPLC-UV chromatogram (280 and 350 nm) (Figure 1 and Figure 2) where the peaks detected were derivatives of galloyl acids (1, 2 and 4), caffeoyl quinic acids (3 and 5), ellagitannins (6–8), ellagic acids (9, 10, 12, 13 and 15) and flavonoid-derivatives (11, 14, 16–23) according to their UV spectra and MS data.

In vitro and ex vitro samples showed similar chromatographic profiles for some compounds, but some differences were detected. The in vitro samples showed five ellagic acid derivatives, while in the ex vitro samples, 13 and 15 were not found. Additionally, the flavonoids 14 and 16–19 detected in ex vitro samples were not found in the in vitro samples (Figure 1 and Figure 2). Furthermore, differences in the content were found between in vitro and ex vitro samples, and among the samples belonging to each group. Nikolova et al. [39] described that the methanol extracts of the samples from ex vitro and in vivo grown *A. montana* plants had significantly higher radical scavenging activity and polyphenolic content than the extracts of in vitro samples. The observed differences in the contents of these biologically active compounds were related to the different growth conditions and stages of plant development. The established biotechnological method of *A. montana* holds promise for the future production of antioxidants.

On the other hand, the work carried out by Giri et al. [40] with suspension culture of *H. edgeworthii* callus describe an efficient method to produce phenolic compounds. In this study, HPLC analysis showed a high content of gallic acid in the in vitro samples (143.63 mg 100 g ^−1^ DW) compared to the wild tuber (5.5 mg 100 g^−1^ DW).

According to the results of HPLC-DAD-ESI-MS^n^, the expression of phenolics compounds was more related to the type of leaf than to the season of the year. It is well-known that phenols are a distinctive feature of *A. chilensis*, and some of its nutritional and pharmacological effects can be attributed to their presence in the plant [17,41].

The first description of phenolic compounds in maqui leaves was carried out by Muñoz et al. [18], who developed a study with fractions of maqui leaf extracts, using different solvents: HE (n-hexane), DCM (dichloromethane), ME (methanol), INFU (infusion, water) and ALK-MIX (DCM + ME) to determine its anti-inflammatory, analgesic and antioxidant activities. The phenolic compounds mainly identified by these authors with HPLC-ESI-MS were present in the ME and INFU fractions. Their study included quercetin 5,3′-dimethyl ether, quercetin 3-*O*-β-D-glucoside and kaempferol, detected in the ME fraction, and caffeic acid and ferulic acid in the INFU fraction. Additionally, ursolic acid, friedelin and quercetin 5,3′-dimethyl ether were identified in the DCM fraction.

Furthermore, Vidal et al. [42] developed a study for the microencapsulation of maqui leaf extracts and identified the presence of phenolics acids (54.36%), flavonoids (42.10%) and stilbenes (3.55%). The authors identified and quantified gallic acid (47.55%), followed by catechin (21.75%) and pelargonidin (14.45%), as the major compounds found in the maqui leaf extracts. The phenolic acids identified by the authors include gallic acid and coumaric acid; however, these compounds were not identified in the current study.

Similarly, the identification of luteonin matched with the results described in more recent studies carried out on maqui leaves by Céspedes et al. [19]. Additionally, they identified, in different fractions of leaves extracts, phenolic compounds such as quercetin, myricetin, rhamnetin, quercitrin, rutin, apigenin, luteonin, *p*-coumaric acid and benzoic acid. The fractions consisted in successive macerations, in the first place, with water and methanol (6:4), followed by organic solvents, from the most polar to the least polar solvents. The results described by Céspedes et al. [19] are similar to those obtained from the qualitative analysis carried out in the present study.

In addition, González-Villagra et al. [43] described quantitative differences in the content of rutin, coumaric acid, ferulic acid and quercetin in young and adult plants under drought stress. Their assays consisted of evaluation of the level of total anthocyanins and ABA regulation in plant response to the application of ABA exogenous and an inhibitor of ABA (Fluridone). The HPLC-DAD analysis carried out on maqui leaves showed the main presence of rutin, independent of the drought stress or ABA and Fluridone application. When compared with this study, coumaric and ferulic acids were not found in their analysed samples, although another two compounds were identified in all their samples.

### 2.3. Quantitative Analysis of the Maqui Leaves

The family of compounds identified in different extracts of the present study, as well as their concentrations, are listed in Table 3, and details of the 23 compounds quantified are shown in Table S1. It is important to highlight that total phenolic content in the in vitro samples was higher than those found in the ex vitro samples. Particularly in the family of galloyl acid derivatives, caffeoyl quinic acid, ellagic acid derivatives and ellagitannins. Moreover, basal spring leaves exhibited a slightly higher level of galloyl acid derivatives, caffeoyl quinic acids and ellagitannins with respect to apical spring leaves, while the basal winter leaves had lower concentrations of these compounds.

In this study, gallic and chlorogenic acids were used as the standard to quantify the family of galloyl quinic and caffeoyl quinic acid derivatives, respectively. Rivera-Tovar et al. [44] analysed maqui leaves by performing extractions in methanol and aqueous acetone, and quantified gallic acid (0.64 ± 0.02 mg g^−1^ DW) and chlorogenic acid (1.44 ± 0.02 mg g^−1^ DW). In the present study, the gallic acid levels were higher in the spring leaves and the in vitro leaves; additionally, the level of chlorogenic acid was higher in the in vitro leaves compared to that obtained by Rivera-Tovar et al. [44]. On the other hand, many phytochemical studies on the composition of maqui berries have been carried out [11]. More recently, Sandoval et al. [45] described and quantified the presence of delphinidin-3-*O*-sambubioside-5-*O*-glucoside (19.645 ± 0.788 mg g^−1^ DW), delphinidin-3-*O*-sambubioside (17.770 ± 1.178 mg g^−1^ DW), cyanidin-3-*O*-sambubioside-5-*O*-glucoside (2.447 ± 0.063 mg g^−1^ DW), cyanidin-3-*O*-glucoside (2.148 ± 0.158 mg g^−1^ DW) and cyanidin-3-*O*-sambubioside (2.642 ± 0.201 mg g^−1^ DW), using UPLC-DAD. In addition to the UHPLC techniques applied to maqui berries, in a recent study carried out by Chen et al. [46], 18 compounds were identified by UHPLC-Q exactive orbitrap-HRMS. They also demonstrated the photoprotective effect of the hydroethanolic maqui berry extract compared to UV-B induced in vitro and in vivo. They verified the protective effect of photodamage of gallic ellagic and protocatechuic acids, and granatin B. Additionally, quercetin derivatives and other delphinidin derivates have been proven to provide skin photoprotection due to their potent antioxidant activities. Moreover, in another study carried out recently by Rodríguez et al. [10] using unripe maqui fruits, the presence of granatin B, kaempferol, quercetin, delphinidin, cyanidin glucosides and other compounds was detected. It is necessary to highlight that granatin B, which is present in fruits, has not been described before in maqui leaves, until the present study. Granatin B is an ellagitannin compound which usually contains the pomegranate fruits, and its functions are associated with anti-inflammatory effects [31]. Gironés-Vilaplana et al. [47] quantified granatin B (0.53 ± 0.11 mg 100 g^−1^ DW) and ellagic acid hexoside (2.01 ± 0.15 mg 100 g^−1^ DW) in maqui fruit extracts carried out with methanol and water (70:30 *v*/*v*). The content of granatin B described by these authors was lower than that of the present study carried out using maqui leaves. However, the content of ellagic acid hexoside described by Gironés-Vilaplana et al. [47] was higher than that found in maqui leaves in the present study (Table S1). Moreover, Genskowsky et al. [48] identified and quantified phenolic compounds, such as ellagic acids (0.94 ± 0.01 mg g^−1^ DW), in maqui berry which were higher than those found in the in vitro leaves of the present study (Table S1). In addition, these authors also quantified rutin (0.20 ± 0.01 mg g^−1^ DW), obtaining similar levels to those found in the basal winter leaves of the present study (Table 3). Finally, the presence of the tetrahidroxyflavone derivatives in maqui leaves has not been previously confirmed in any study carried out on this plant species.

According to HPLC-MS identification, *A. chilensis* leaves showed the presence of quercetin, catechin, kaempferol and 3-caffeoyl quinic acid. With regards to sample quantification, the compounds 3-caffeoylquinic acid and catechin showed the highest concentrations, followed by kaempferol. The quercetin was only detected in ex vitro maqui leaves (Table 4).

These results agree with those of several authors who reported the presence of quercetin, catechin and kaempferol in maqui leaves [18,19,42,43]. The values of quercetin (7040 μM) and catechin (6669.24 μM) obtained in the ex vitro samples were higher than those obtained by Vidal et al. [42] (quercetin (10.92 μM) and catechin (182.59 μM)). The presence of caffeolyl quinic acid is very similar to that reported by Gironés-Vilaplana et al. [47,49], who mentioned that caffeoyl quinic acid derivatives were present in fruits. In addition, Rivera-Tovar et al. [44] realized that the successive extractions of maqui leaves in methanol and aqueous acetone showed higher values than compounds found in this study from HPLC-MS analysis. The authors used UPLC-MS for the identification and quantification of compounds such as quercetin (1.35 ± 0.03 mg g^−1^ DW), catechin (2.25 ± 0.03 mg g^−1^ DW), kaempferol (0.90 ± 0.01 mg g^−1^ DW) and 3- caffeoyl quinic acid (1.44 ± 0.02 mg g^−1^ DW).

**Figure 6:**
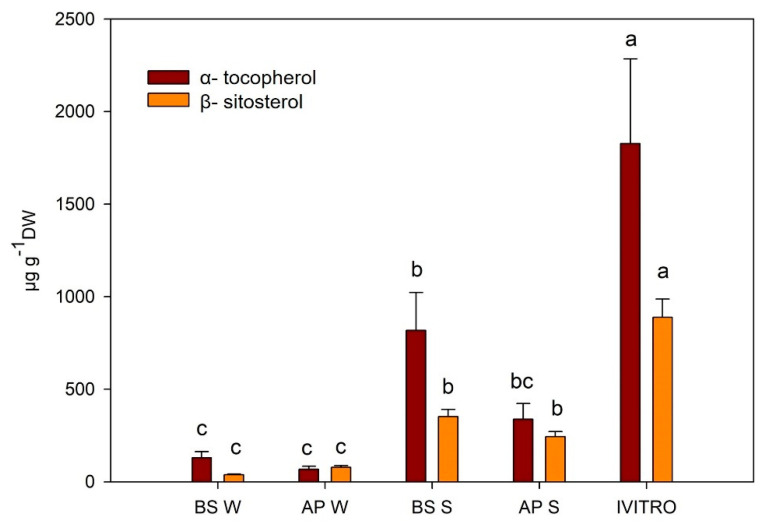
Yield (µg g^−1^ DW) of α-tocopherol and β-sitosterol from *A. chilensis* leaves. Median ± E.E. (one-way ANOVA, Tukey, *p* ≤ 0.05, *n* = 3). Different letters in the same row indicate significant differences according to Tukey´s test. BS W: Basal winter leaves, AP W: apical winter leaves, BS S: basal spring leaves, AP S: apical spring leaves, and IVITRO: in vitro leaves.

**Figure 7:**
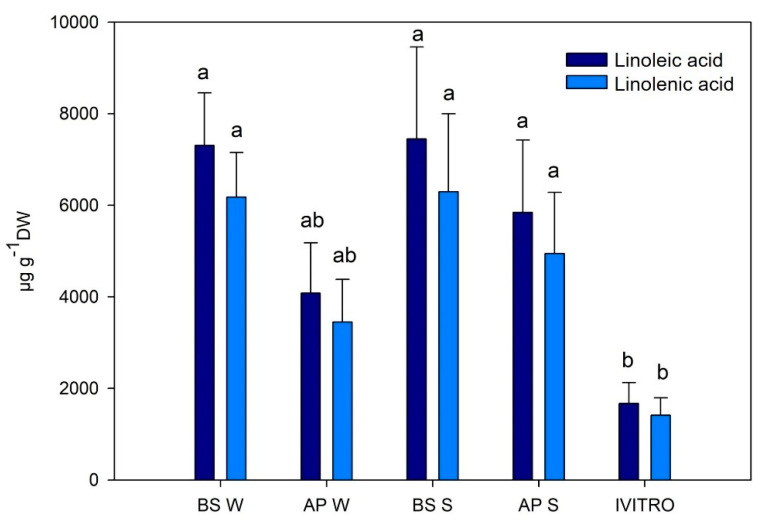
Yield (µg g^−1^ DW) of linoleic and linolenic acids from *A. chilensis* leaves. Median ± E.E. (one-way ANOVA, Tukey, *p* ≤ 0.05, *n* = 3). Different letters in the same row indicate significant differences according to Tukey´s test. BS W: Basal winter leaves, AP W: apical winter leaves, BS S: basal spring leaves, AP S: apical spring leaves, and IVITRO: in vitro leaves.

GC-MS is one of the most reliable biophysical methods due to its specificity and repeatability; this was utilized for the phytochemical profiling of *A. chilensis* leaves. The following lipophilic compounds, e.g., β-sitosterol, α-tocopherol (Figure 6), linoleic and linolenic (Figure 7) was observed. The compounds were quantified using external commercial patterns. The mass spectra and the structure of β-sitosterol, α-tocopherol and linoleic and linolenic acid are found in the Supplementary Material Figures S1 and S2, respectively. The highest values of β-sitosterol and α-tocopherol were obtained from the in vitro leaves, followed by spring BS leaves. Furthermore, the lowest values for β-sitosterol and α-tocopherol were obtained in winter leaves. In contrast, the higher values for linoleic and linolenic acid were obtained in ex vitro leaves as compared to the in vitro leaves of *A. chilensis*. It is important to note that α-tocopherol, linoleic acid and linolenic acid have not been described and quantified in maqui leaves before. The level of α-tocopherol described by Quispe-Fuentes et al. [50] in maqui fruit (31.7 ± 0.5 µg g^−1^ DW) was low when compared with the leaves. Additionally, the authors described the presence of palmitic acid (9.79 ± 0.16 g 100 g^−1^ of the sample), linoleic acid (44.63 ± 0.25 g 100 g^−1^ of the sample) and linolenic acid (2.24 ± 0.23 g 100 g^−1^ of the sample) in maqui fruit. The first quantification of phytosterol was described by Muñoz and Ramos [51]. They identified the main phytosterol as β-sitosterol, followed by campesterol, sitostanol and campestanol. They described the value of β-sitosterol as 4.3 ± 1.0 µg g^−1^ DW. The α-tocopherol and β-sitosterol metabolites were produced by plants at low concentrations, and their production increased in the in vitro leaf samples (Figure 6). In this study, the highest values of linoleic and linolenic acids were obtained in BS maqui leaves (Figure 7).

The TFC of the different extracts of *A. chilensis* is shown in Table 5. BS winter extract had the highest TFC, followed by those of apical leaves in spring and BS in spring. No significant differences were observed between them. The lowest value of TFC was obtained in the extracts of in vitro leaves.

Plants of maqui have high amounts flavonoids and potent antioxidant activity, leading to various defensive and disease-fighting properties [11]. Phenolic compounds are a plant’s secondary metabolites, considered as fundamental plant constituents due to the presence of one or more hydroxyl groups on their aromatic ring. The results of TFC are similarly to those described by Vidal et al. [42] for those obtained in the in vitro leaves (0.061 ± 0.01 mg RE mL^−1^). The assay was realized with autumn leaves and not described with a difference state of development of the leaf. It is likely that the difference in the extract method is the major influence on the content of flavonoids. The amount of flavonoid of the methanolic extract was approximately higher than that of the hydroethanolic extracts proposed by the author.

## 3. Materials and Methods

### 3.1. Plant Material

Maqui leaves were collected between August and December 2017 at the University of Concepción, Biobío Region, Chile. The samples were collected in the winter and spring seasons. Adult male maqui plants reaching a height of 3 m were used. In them, two types of samples were collected: leaves of the upper third of the branch, called apical (AP), and leaves of the lower third of the branch, called basal (BS).

#### 3.1.1. In Vitro Plant Cultures

In vitro plant cultures of maqui were initiated using vegetative nodal segments as initial explants. This plant material was obtained from mother plants collected in November 2018. The vegetative samples were disinfected with ethanol 70% (1 min), and then with a hypochlorite solution (0.5%) containing Tween^®^ 20 detergent (final concentration of 0.01%, *v*/*v*) for 10 minutes. After the disinfection time, the nodal explants were washed 4 times with sterile distilled water. After that, they were placed in glass tubes with 20 mL of culture medium. The culture medium contained half-strength Murashige and Skoog [52] basal mineral medium, vitamins, sucrose (20 g L^−1^), polyvinylpolypyrrolidone (0.5 g L^−1^), kinetin (0.5 mg L^−1^) and naphthalene acetic acid (0.05 mg L^−1^). The pH was then adjusted to 6.0 with NaOH 1N or HCl 1N, and phytagel (2.7 g L^−1^) was added. Shoots were developed from these nodal segments, and sub-cultured every 25 days. In vitro plants were grown at a 16-h photoperiod (photosynthetic photon flux density (PPFD) of 40–60 µmol photons m^−2^ s^−1)^, and a temperature of 25/20 °C, day/night, respectively.

#### 3.1.2. Preparation of the Samples from In Vivo and In Vitro Maqui Leaves

For phytochemical analysis, samples from adult maqui plants constituted by fresh AP and BS leaves (40 g) were dried at 37 °C for two days. Dried leaves were crushed to obtain a fine powder and then macerated by exhaustion in methanol-HCl 0.1%. The total extract was concentrated at 37 °C and lyophilized for 24 h. In addition, for preparation of samples from in vitro plant material, leaves from in vitro maqui plants were collected (2 g) and lyophilized for extract preparation. The lyophilized material was macerated with methanol-HCl (0.1%) for 24 h and then centrifuged, and the supernatant was then collected. It was dried at 37 °C and frozen at −20 °C until use.

### 3.2. Determination of Polyphenolic Compounds by HPLC-DAD-ESI (Ion Trap)-MS^n^

Chromatographic separations of different components of the extracts were carried out in a Mediterranean Sea C18 column (150 × 4.60 mm, 3 µm particle size, Teknokroma, Spain) at room temperature, as described by Ferreres et al. [53], with minor modifications. Elution was performed with a mobile phase of two solvents, water-formic acid (1%) (A) and acetonitrile (B), starting with 10% B and using a gradient to obtain 25% B at 30 min and 70% B at 35 min at a flow rate of 0.8 mL min^−1^ and an injection volume of 20 µL. The system was HPLC (Agilent model 1200) coupled to a DAD and a mass spectrometer with ion trap technology (Bruker, model Amazon, Ultra High-Speed ion trap).

Chromatograms were recorded at 280 and 350 nm, and the MS system was operated in negative ion mode using the MS/MS Fragmentation Amplitude 1.00 V (mass fragmentation energy ramp), mass range *m*/*z* 100–1200. For quantitative analysis, external standard calibration curves for ellagic acid (assay ≥ 95%), rutin (assay ≥ 95%), chlorogenic acid (assay ≥ 99%) and gallic acid (assay ≥ 99%), all purchased from Sigma-Aldrich (Steinheim am Albuch, Germany), were used. The calibration curves of the external standards were: ellagic acid (y = 70x − 131.7; R^2^ = 1); rutin (y = 22.822x − 14.77; R^2^ = 1); chlorogenic acid (y = 15.109x − 86.336; R^2^ = 0.9994); gallic acid (y = 10.618x + 130.54; R^2^ = 0.9931). The range of each standard curve was 3.125–1000 μM. The results were expressed in µg g^−1^ DW (dry weight).

### 3.3. Determination of Polyphenolic Compounds by HPLC-MS

BS leaves collected in spring and in vitro leaves were analysed by an HPLC-MS system (Agilent Series 1200, Agilent Technologies, Santa Clara, CA, USA), as described by Sánchez-Pujante et al. [54]. Separation was performed at room temperature on a C18 column (4.6 mm × 250 mm, 5 μm). The mobile phase consisted of solvent A (formic acid 0.5%) and solvent B (acetonitrile-formic acid 0.1%), using a gradient described as follows: 0 min, 2% solvent B; 10 min, 20% solvent B; 36–37 min, 100% solvent B; 37.5 min, 2% solvent B; 40 min, 2% solvent B. The flow rate was 0.8 mL min^−1^, and the injection volume was 20 μL. Mass spectral analysis was carried out using a TOF/Q-TOF MS (Agilent Series 6220, Agilent Technologies, USA) equipped with an ESI operating in negative ion mode. The operation parameters were: capillary, fragmentor and octopole RF voltages were 2500, 180 and 250V, respectively; nebulizer pressure, 60 psi; drying gas flow, 12 L/min; drying gas temperature, 350 °C. Mass range was 50–1200 *m*/*z* and scan rate was 1.9 spectra/sec. External standard calibration curves for catechin (assay ≥ 99%), 3-0-caffeoylquinic acid (assay ≥ 98%), quercetin (assay ≥ 95%) and kaempferol (assay ≥ 97%), all purchased from Sigma-Aldrich (Germany), were used. The calibration curves of the external standards were: catechin (y = 148,394x + 1500.1; R^2^ = 0.99); 3-caffeoylquinic acid (y = 102,069x + 5719.1; R^2^ = 0.9997); quercetin (y = 2,198,650.63x + 946,539.58; R^2^ = 0.98); kaempferol (y = 2,946,294.44 + 965,232.12; R^2^ = 0.99). The range of each standard curve was 0.1–1 μg mL^−1^.

### 3.4. Determination of Lipophilic Compounds by GC/MS

Samples were analysed by GC/MS, as described by Sabater-Jara et al. [55]. The identification of metabolites was based on the mass spectra (EI, 70 eV) obtained from a gas chromatograph (Agilent Technologies 6890 Network GS System) equipped with a mass selective detector (Agilent Technologies 5973). A 30 m × 0.25 mm × 0.25 µm capillary column (Agilent 19091 S−433HP−5MS) was used for GC/MS analysis. The GC oven temperature was programmed from 60 to 310 °C at 10 °C/min for the analysis of the metabolites. A constant flow rate of 1 mL min^−1^ was set using helium as a carrier gas. The injection volume was 1 µL. The mass range was recorded from *m*/*z* 50 to 800. Data was obtained in scan mode using electron impact ionization.

The identification of maqui metabolites was conducted by comparing the experimental mass spectra with the National Institute Standard and Technology (NIST) spectral library. Likewise, the metabolites were identified and quantified by comparing with respective retention times and mass spectra from external standards. External standard calibration curves for α- tocopherol (assay ≥ 96%), β- sitosterol (assay ≥ 95%), linoleic acid (assay ≥ 99%) and linolenic acid (assay ≥ 99%), all purchased from Sigma-Aldrich (Germany), were used. The calibration curves of these external standards were: α-tocopherol (y = 660,602.95x − 8,437,255.35; R^2^ = 0.99); β- sitosterol (y = 486,377,151.06x − 4,380,294.85; R^2^ = 0.99); linoleic acid (y = 64,142.56x − 18,230,407.54; R^2^ = 0.99); linolenic acid (y = 72,376.19x − 1,546,576.93; R^2^ = 0.99). The range of each standard curve was 0.1–10 μg mL^−1^.

### 3.5. Total Flavonoid Content

Total flavonoid content (TFC) was measured by the aluminium chloride assay described by Ahmed et al. [56], with some modifications. All samples were analysed in triplicates. An aliquot of 0.2 mL of extracts or standard rutin solution (0.02–0.4 mg L^−1^) was added to 0.8 mL methanol (50%). Then, 60 μL aqueous sodium nitrite solution (0.5 M) was added, followed by 60 μL aluminium chloride solution (0.3 M). After 5 min, 0.4 mL sodium hydroxide solution (1 M) was added. After that, the content was mixed well before measuring its absorbance at 506 nm on a UV visible spectrophotometer against a blank. TFC was expressed as rutin equivalents (RE) per mL of extract (mg RE mL^−1^).

## 4. Conclusions

The HPLC-DAD-ESI (Ion Trap)-MS^n^ analysis showed the presence of different phenolic compounds in both apical and basal adult (20 compounds), and in vitro maqui leaves (16 compounds). The compounds identified were classified into the groups of galloyl and caffeoyl quinic acids, ellagitannins, ellagic acid derivatives and flavonoid derivatives. In addition, the HPLC-MS analysis indicated that the extract from BS spring leaves included quercetin, catechin, kaempferol and 3-caffeoyl quinic acids compounds, while in the in vitro leaves extract, no quercetin was present. Determination of lipophilic compounds was performed using GC/MS. The samples of in vitro leaves showed a high presence of α-tocopherol and β-sitosterol. In contrast, the samples of adult leaves presented a high level of linolenic and linoleic acids. The results of the current study suggest that maqui leaves could be an excellent source of antioxidants and lipophilic compounds for many industries such as the nutraceutical and pharmaceutical industries. Likewise, this study is preliminary to future research on the production of plant secondary metabolites through the use, not only of the in vitro culture of *A. chilensis* plants by using temporary immersion systems, but also to establish maqui cell suspension cultures which, under elicitation, constitute an efficient strategy to provide secondary metabolites with a significant impact on human health.

## Figures and Tables

**Table 1:** Rt, Molecular Formula, [M-H]^−^ and MS2 [M-H]^−^ data for the ellagic acid derivatives detected in methanolic extracts of *A. chilensis* leaves ^1^.

Compounds ^2^		Rt (min)	[M-H]^−^, *m*/*z*	MS^2^[M-H]^−^, *m*/*z*		
				−132	−146	−162
**9**	Ellag ac-Hex	8.0	463			301
**10**	Ellag ac-Pt	10.3	433	301		
**12**	Ellag ac-Pt	10.7	433	301		
**13**	Ellag ac-Rhmn	11.0	447		301	
**15**	Ellag ac-Rhmn	11.6	447		301	

^1^ Main fragments observed; ^2^ Ellag ac: ellagic acid; Pt: pentoside; Rhmn: rhamnoside; Hex: hexoside.

**Table 2:** Rt, Molecular Formula, [M-H]^−^ and MS^2^[M-H]^−^ data for flavonoids derivatives detected in methanolic extracts obtained from *A. chilensis* leaves ^1^.

Compounds ^2^		Rt (min)	UV (nm)	[M-H]^−^, *m*/*z*	MS^2^[M-H]^−^, *m*/*z*	
					−152	[Aglc-H]^−^
**11**	Qct-3-(Gall)Hex	10.5	268, 288sh, 352	615	463 (100)	301 (25)
**14**	Qct-3-(Rhmn)Hex	11.0	255, 266sh, 295sh, 354	609		301 (100)
**16**	Qct-3-(Rhmn)Hex	11.2	256, 266sh, 298sh, 355	609		301 (100)
**17**	Qct-3-Hex	11.6	256, 266sh, 298sh, 354	463		301 (100)
**18**	Qct-3-Hex	11.9	256, 266sh, 298sh, 355	463		301 (100)
**19**	Lut-7-Hex	12.0	256, 266sh, 348	447		285 (100)
**20**	Qct-3-Pt	13.1	256, 266sh, 298sh, 355	433		301 (100)
**21**	tetOHFlv-(Rhmn)Hex	13.6	248sh, 268, 290sh, 336	593		285 (100)
**22**	tetOHFlv-Hex	14.3	248sh, 268, 290sh, 336	447		285(100)
**23**	triOH-diOMeFlv-der	14.8	252, 266sh, 298sh, 346	659		329(100)

^1^ Main fragments observed; ^2^ Aglc: aglycon; Gall: gallic acid; Qct: quercetin; Lut: luteolin; tetOHFlv: 5,7,2,4′-tetrahydroxyflavone; triOH-diOMeFlv-der: trihydroxy-dimethoxyflavone-derivative; Pt: pentoside; Rhmn: rhamnosid; Hex: hexoside.

**Table 3:** Polyphenolic compounds quantified in methanolic extracts of *A. chilensis* leaves (µg g^1^ DW).

Sample	Galloyl Acid Derivatives	Caffeoyl Quinic Acids	Ellagitannins	Ellagic Acid Derivatives	Flavonoid Derivatives	Total Polyphenols
AP S	777.77 ± 15.65 c	89.67 ± 0.70 b	3306.80 ± 33.88 b	27.99 ± 0.33 b	894.69 ± 2.67 b	5096.92 ± 37.42 c
BS S	1004.52 ± 10.28 b	87.83 ± 1.30 b	3355.70 ± 27.61 b	36.04 ± 1.45 c	928.35 ± 4.96 a	5412.43 ± 29.94 b
AP W	400.96 ± 5.71 d	21.58 ± 0.14 c	1055.90 ± 7.28 c	8.13 ± 0.10 d	379.24 ± 2.18 d	1865.81 ± 9.51 d
BS W	362.33 ± 9.20 e	6.90 ± 0.09 c	915.33 ± 13.98 c	5.33 ± 0.05 d	230.77 ± 2.04 e	1520.66 ± 16.86 e
IVITRO	4973.71 ± 56.33 a	852.37 ± 17.79 a	4513.45 ± 84.90 a	177.83 ± 2.08 a	523.51 ± 11.42 c	10,611.44 ± 78.41 a

Median ± E.E. (one-way ANOVA, Tukey, *p* ≤ 0.05, *n* = 3). Different letters in the same row indicate significant differences according to Tukey´s test. BS W: Basal winter leaves, AP W: apical winter leaves, BS S: basal spring leaves, AP S: apical spring leaves, and IVITRO: in vitro leaves.

**Table 4:** HPLC-MS data of the identified and quantified compounds from leaves of *A. chilensis* (µg g^−1^ DW).

Leaf Type	Quercetin	Catechin	Kaempferol	3-Caffeloylquinic Acid
In vitro		35.44 ± 3.54 b	6.69 ± 0.67 b	25.93 ± 2.59 b
BS S	145.73 ± 14.57 a	132.59 ± 13.26 a	25.93 ± 2.59 a	253.77 ± 25.38 a

Median ± E.E. (one-way ANOVA, Tukey, *p* ≤ 0.05, *n* = 3). Different letters in the same row indicate significant differences according to Tukey´s test.

**Table 5:** Total flavonoids content (mg rutin equivalents per mL) in ex vitro and in vitro leaves of *A. chilensis* extracts.

Types of Leaves		TFC (mg RE mL^−1^)
1	BS Winter	0.086 ± 0.004 a
2	AP Winter	0.074 ± 0.001 c
3	BS Spring	0.081 ± 0.005 bc
4	AP Spring	0.083 ± 0.002 b
5	In Vitro	0.061 ± 0.002 c

Median ± E.E. (one-way ANOVA, Tukey, *p* ≤ 0.05, *n* = 4). Different letters in the same row indicate significant differences according to Tukey´s test.

## Data Availability

The data presented in this study are available on request from the corresponding author.

## Supplementary Materials

The following are available online at https://www.mdpi.com/article/10.3390/plants11010037/s1, Figure S1. Mass spectra and chemical structure of α-tocopherol (a) and β-sitosterol (b); Figure S2. Mass spectra and chemical structure of linoleic acid (a) and linolenic acid (b); Table S1: Detailed quantified profile of the methanolic extracts obtained from *A. chilensis* leaves (µg g^−1^ DW). Different letters in the same row indicate significant differences according to Tukey´s test.
