# Supplementary material for: Comparative Study of Metabolomic Profile and Antioxidant Content of Adult and In Vitro Leaves of Aristotelia chilensis
Source: Plants (Basel). 2021 Dec 23;11(1):37. doi: 10.3390/plants11010037 (PMC8747544; doi:10.3390/plants11010037)
Supplement: Supplementary file 1 [file plants-11-00037-s001.zip › plants-1469869-supplementary.pdf]

Supplementary Materials

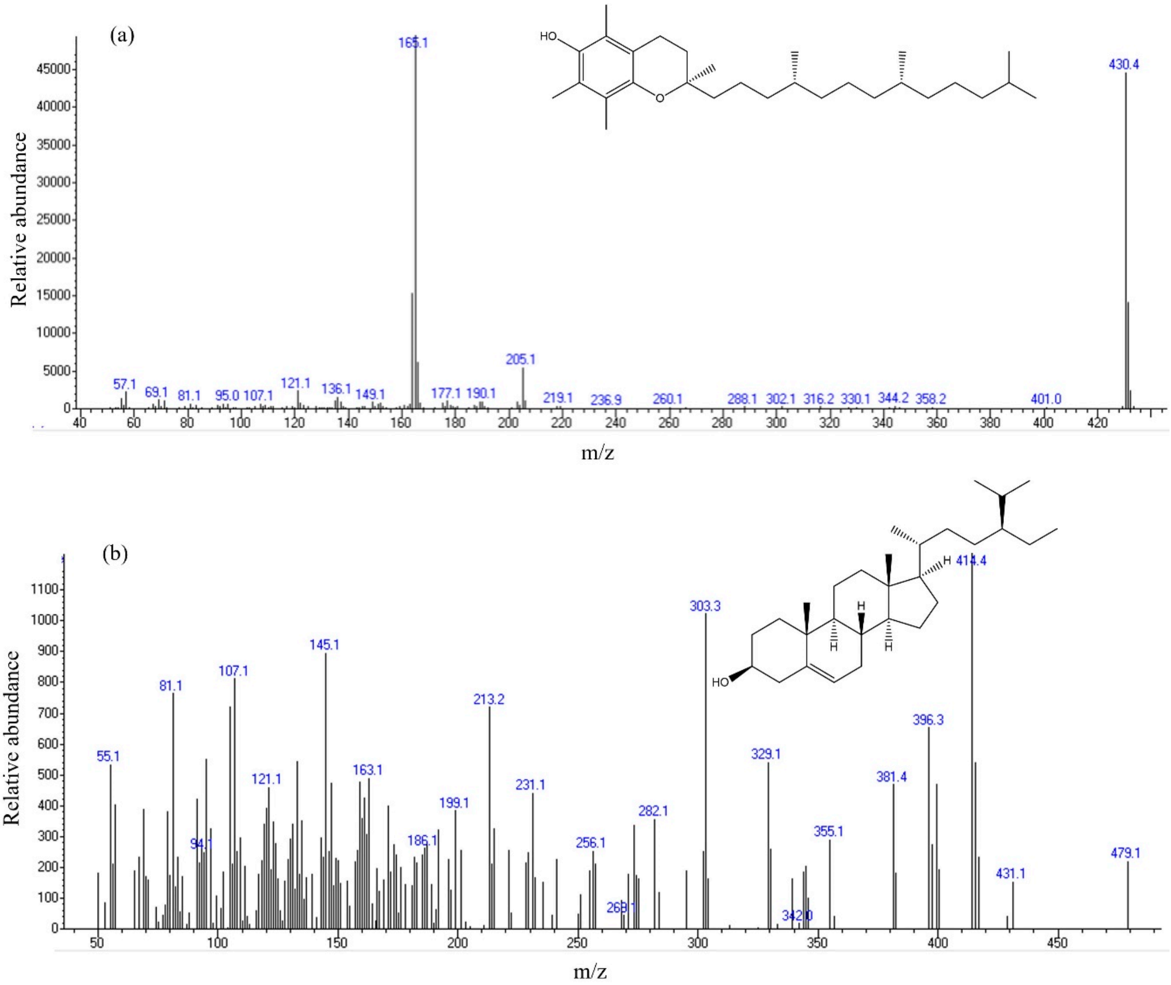

Figure S1. Mass spectra and chemical structure of α-tocopherol (**a**) and β-sitosterol (**b**).

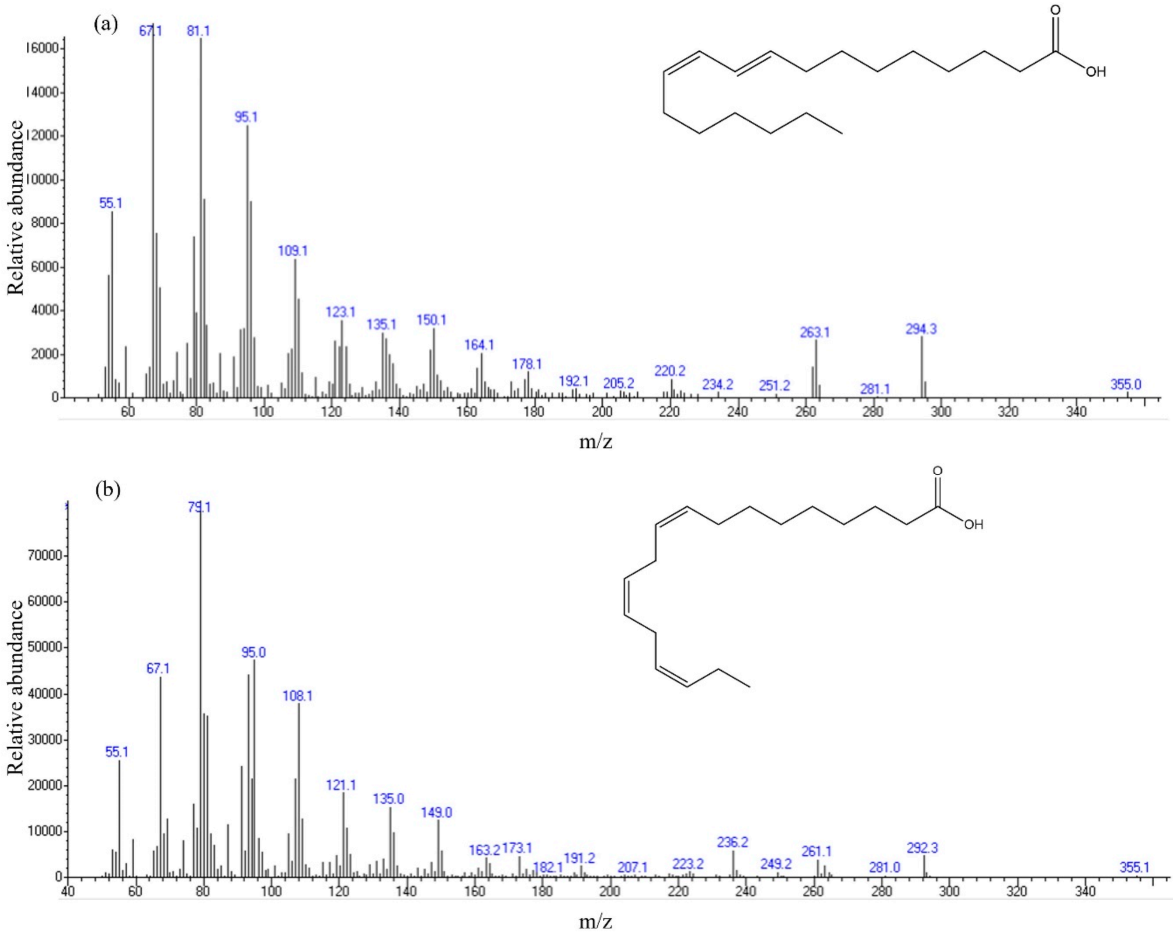

Figure S2. Mass spectra and chemical structure of linoleic acid (**a**) and linolenic acid (**b**).

Table S1. Detailed quantified profile of the methanolic extracts obtained from *A. chilensis* leaves (μg g^{-1} DW). Different letters in the same row indicate significant differences according to Tukey´s test ($p \leq 0.05$).

	Compounds	AP S	BS S	AP W	BS W	IVITRO
Peak	Galloyl acids derivatives					
1	Galloyl-hexoside	250.23 ± 10.35c	400.36 ± 8.21b	145.39 ± 4.40d	68.16 ± 6.12e	2135.17 ± 144.39a
2	Galloyl quinic acid	309.80 ± 9.95b	265.36 ± 5.21c	119.93 ± 2.07d	83.09 ± 2.36d	1571.63 ± 148.94a
4	Di-galloyl quinic acid	217.74 ± 6.25c	338.80 ± 3.32b	135.64 ± 3.00d	211.09 ± 6.46c	880.93 ± 99.31a
	Caffeoyl quinic acids					
3	3-caffeoyl quinic acid	64.89 ± 0.57b	58.32 ± 1.29b	15.36 ± 0.14c	5.04 ± 0.05c	791.28 ± 20.87a
5	4-caffeoyl quinic acid	24.78 ± 0.40c	29.51 ± 0.15b	6.21 ± 0.02d	1.86 ± 0.08e	44.73 ± 0.95a
	Ellagitannins					
6	Ellagitannin	125.02 ± 5.06c	148.91 ± 1.15b	20.06 ± 0.23d	19.19 ± 0.34d	273.34 ± 24.98a
7	Granatin B	1644.52 ± 33.88b	1338.17 ± 18.10c	563.66 ± 5.93d	465.13 ± 13.07e	1824.00 ± 65.61a
8	Ellagitannin	1537.27 ± 14.89c	1868.62 ± 20.83b	472.17 ± 4.22d	431.01 ± 4.96d	2444.44 ± 74.18a
	Ellagic acid derivatives					
9	Ellagic acid-Hexoside	14.05 ± 0.31b	11.29 ± 1.42c	2.82 ± 0.01d	2.10 ± 0.01d	18.61 ± 0.91a
10	Ellagic acid-Pentoside		11.69 ± 0.28b			42.96 ± 3.78a
12	Ellagic acid -Pentoside	13.94 ± 0.12b	13.06 ± 0.11c	5.32 ± 0.10d	3.23 ± 0.05e	20.22 ± 3.31a
14	Ellagic acid-Rhamnoside					52.15 ± 5.86
17	Ellagic acid					26.39 ± 5.54
	Flavonoid derivatives					
11	Quercetin-3-(gallic acid)Hexoside	117.72 ± 0.59b	135.29 ± 0.57a	34.00 ± 0.69c	20.13 ± 0.30d	
13	Quercetin-3-(2-Rhamno-side)Hexoside	37.11 ± 0.66a	36.73 ± 0.19a	19.83 ± 0.18b	10.36 ± 0.28c	
15	Quercetin-3-(6-Rhamno-side)Hexoside	77.90 ± 0.43a	68.39 ± 0.26b	39.93 ± 0.60c	22.71 ± 0.38d	
16	Quercetin-3-Hexoside	152.18 ± 1.11b	165.64 ± 2.26a	59.70 ± 0.69c	26.52 ± 0.57d	
18	Quercetin-3-Hexoside	44.71 ± 1.24b	47.14 ± 1.09a	22.02 ± 0.34c	10.59 ± 0.18d	
19	Quercetin-3-Rhamnoside	45.45 ± 0.78a	40.83 ± 0.54a	7.53 ± 0.37c	7.67 ± 0.17b	
20	Quercetin-3-Pentoside	42.54 ± 0.43b	43.51 ± 0.16a	9.46 ± 0.06c	2.14 ± 0.34d	
21	Tetrahydroxyflavone-(6-Rhamnoside)Hexoside	143.82 ± 1.10b	130.57 ± 1.04c	64.45 ± 0.40d	49.85 ± 1.02e	224.85 ± 12.55a
22	Tetrahydroxyflavone-Hexoside	194.94 ± 1.06b	225.90 ± 4.03a	112.68 ± 1.72c	73.48 ± 1.52d	220.75 ± 23.91a
23	Trihydroxy-dimethox-yflavone	38.31 ± 0.48b	34.35 ± 0.51c	9.62 ± 0.09d	7.32 ± 0.09e	40.01 ± 3.33a

BS W: Basal winter leaves, AP W: apical winter leaves, BS S: basal spring leaves, AP S: apical spring leaves, and IVITRO: in vitro leaves.
